# A Perception-Augmented Hidden Markov Model for Parent–Child Relations in Families of Youth with Type 1 Diabetes

**DOI:** 10.1007/s12561-022-09360-8

**Published:** 2022-12-30

**Authors:** Ruijin Lu, Tonja R. Nansel, Zhen Chen

**Affiliations:** 1 Division of Population Health Research, Eunice Kennedy Shriver National Institute of Child Health and Human Development, National Institutes of Health, Bethesda, MD 20892, USA

**Keywords:** Hidden Markov model, Multivariate mixed model, Parent–Child relationship, Type 1 diabetes

## Abstract

In youth with Type 1 diabetes, adherence to medical treatment regimens requires the involvement of both parent and child. A clinic-integrated behavioral intervention in the Family Management of Diabetes (FMOD) trial was shown to be effective in controlling deterioration in glycemic level; yet the mechanism remains unknown. It is possible that the effectiveness is through improved Parent–Child relation. To investigate whether the intervention improves Parent–Child relations, we proposed a novel approach that allows differential perceptions of parent and child toward the unobserved Parent–Child relationship. Leveraging manifesto data collected from both parent and child in the FMOD trial, the proposed approach extended a standard hidden Markov model by inserting a layer of parent- and child-specific hidden states. We took a Bayesian perspective to estimation and developed an efficient computational algorithm to sample from the joint posterior distribution. Extensive simulations were conducted to demonstrate the performance of the proposed modeling framework. Application to the FMOD trial data reveals that families in the intervention arm are more likely to stay in the Harmonious Parent–Child relation state and less likely to transition from Harmonious to Indifferent state. Compared to parent, child tends to have a more heterogeneous perception of the Parent–Child relation.

## 1 Introduction

Adherence to the medical treatment regimens is important in effective management of type 1 diabetes (T1D). In youth with T1D, good adherence requires well-functioning partnership of both the parent and the child in checking blood glucose levels, administering insulin, and monitoring diets and physical activities. Unfortunately, during pre-adolescence and adolescence, these burdensome daily demands coincide with a vulnerable period when youths undergo social, emotional, and physical changes that challenge Parent–Child relations [1, 2]. Glycemic control typically worsens during this period [3, 4], which can have both short- and long-term adverse effects. Pre-adolescence and adolescence also provide a critical window of opportunity for youth with T1D to develop self-care skills that can positively project into adulthood and lead to improved long-term outcomes. It is, therefore, of high public health importance to develop tools and strategies that can help parents and youths better adhere to their management regimen and improve glycemic control.

A wide range of behavioral interventions targeting diabetes-related Parent–Child relations have been evaluated [5–14]. The NICHD Family Management of Diabetes (FMOD) clinical trial [15] investigated a practical, low-intensity, and clinic-integrated behavioral intervention. Briefly, the FMOD trial enrolled and randomized 390 families of youth with T1D into either an intervention or a usual-care treatment arm at baseline. These families were followed for approximately 2 years, with intervention contacts and data collection occurring at routine clinic visits (typically every 3–4 months). A description of the trial design and intervention conditions of the FMOD trial is provided in Sect. 2; more detailed information can be found in Nansel et al. [16]. The intervention was effective in reducing deterioration in glycemic control, registering a statistically significantly smaller increase in HbA1C from baseline to 24 month compared to the usual-care arm [16]. Some important secondary questions remain, however. What is the mechanism through which the study intervention worked to control the child’s HbA1C level? Is this effectiveness a result of improved Parent–Child relationship? In this paper, we address these questions by investigating whether the intervention affects Parent–Child relations.

In FMOD trial, the Parent–Child relationship is manifested by constructs previously shown to be relevant to diabetes management, two of which are considered in this paper. The first, parent task involvement, is assessed using the Diabetes Family Responsibility (DFR) Questionnaire [17] which includes 17 items querying the division of responsibility between the child and the parent for diabetes management tasks. The second, Parent–Child conflict (PCC), indicates the level of family conflict over diabetes management issues using the 19-item Diabetes Family Conflict Scale [18]. In the remainder of the paper, these two constructs will be referred to as DFR and PCC, respectively. The parent and the child completed identical versions of both questionnaires at each study visit, giving rise to a longitudinal dyadic data structure. A summary score for each construct at each visit was calculated as the sum of responses to all items. The four summary variables, DFR and PCC reported by the parent and the child, respectively, formed the outcome data in our analysis and will be referred to as “manifesto variables” hereafter.

Since the manifesto variables are driven by the unobserved Parent–Child relation, their marginal distributions are potentially multi-modal (see Fig. A.1). Consequently, a hidden Markov model (HMM) is more advantageous than a Markov model in describing the underlying mechanism. As a generalization of finite mixture model under the longitudinal setup, a HMM can describe over-dispersion in data naturally. Moreover, modeling the unobserved relation as a Markov chain assumes that the state of Parent–Child relation is dynamic. It helps us to interpret how the relation evolves over time by estimating the Markov chain’s transition matrix. Furthermore, mixed hidden Markov models [19, 20] permit greater flexibility in modeling correlation structure by introducing random effects in and allowing multivariate manifesto variables. By estimating transition matrices for different treatment arms, we can identify one subgroup of the population that is most responsive to the intervention. Appropriate statistical inferences can be conducted to examine whether the intervention impacts how the families transition from one Parent–Child relationship state to another. This approach is not satisfactory, however, as it implicitly assumes that the parent and the child are homogeneous in the process of manifesting the hidden states. This assumption is likely unreasonable and fails to recognize that the perception of the child may be substantially different from that of the parent. Indeed, when we fit separate HMMs to parents’ and children’s data, respectively, we obtained very different results (see Sect. 2). However, treating the parent’s constructs separately from the child’s will result in loss of information. Moreover, the disjoint model estimations make it difficult to interpret the results.

In this paper, we propose a novel extension of HMM, called perception-augmented hidden Markov model (pHMM), which jointly models parent’s and child’s manifesto variables while explicitly allows each member to have heterogeneous perceptions toward Parent–Child relations. Allowing differential perceptions helps us answer the research question without the above pitfalls, while at the same time having a solid theoretical underpinning.

Developmental psychology has long recognized the importance of perceptions by the parent and the child [21]. For example, Van Slyke and Leton [22] examined how perception of family relations in children affects their school adjustment. Palmer et al. [23] reported that perceptions from mother, father, and children can be related to demographic factors in the context of diabetes control. It is, therefore, desirable to be able to model how Parent–Child relations transition over time and how they are perceived differentially by members of the dyad (i.e., a pair of parent and child). To that end, our proposed framework will assume the existence of a hidden state of family relationship, which forms a first-order Markov process. Based on this family relationship state, the parent and the child each form their perceived Parent–Child relationship state through a perception matrix. The observed outcomes from parent or child are simply manifestations of their corresponding latent perceived states. In addition to allowing the treatment arm-specific transition matrices, the proposed pHMM can estimate perception matrices that are specific to the parent and the child, as well as to the study arms. As a result, pHMM can provide insights into some additional research questions: (1) Does the intervention affect the dynamic of parent–child relations? (2) Do the parent and the child perceive their relationship differently? (3) Are perceptions different between treatment arms? We believe this new modeling framework suits the FMOD research questions better than the standard HMM approaches.

As a mixture model with the mixing distribution a finite-state Markov chain, the HMM has been widely used in many applications including smoking cessation and alcoholism treatment [24], education [25], and psychiatry [26], among many others. Central to a successful implementation of the classical HMM is the Baum–Welsch algorithm that used forward and backward recursion [27, 28] and the Viterbi algorithms [29]. HMMs have been extended to longitudinal data settings for modeling multiple processes simultaneously [19, 20], to hierarchical and nested HMMs [30], to mediation analysis and causal inference framework [26, 31]. We incorporate random effects similar to mixed Hidden Markov models (MHMM) [19, 20].

The paper is organized as follows. We motivate the proposed approach by discussing some preliminary analyses of the FMOD trial data in Sect. 2. We then describe the proposed perception-augmented HMM in Sect. 3 and its estimation in Sect. 4. In Sect. 5 we apply pHMM to the FMOD trial data, interpret the results, and check model goodness of fit. To evaluate the performance of pHMM, we conduct extensive simulations in Sect. 6. Finally, in Sect. 7, we conclude with discussions on strengths and weaknesses of the proposed approach and provide thoughts on future work directions.

## 2 An Initial Look at the FMOD Trial Data

The FMOD trial recruited 390 families of youth with T1D and randomly assigned them to either an intervention (n=201) or a usual-care (n=189) arm at baseline. Each family was followed for 2 years or until dropout. Brief questionnaires were administered at each clinic visit (typically every 3–4 months). In addition, a telephone assessment at study midpoint and an in-clinic 24-month final assessment were conducted. Following Nansel et al. [16] and Temmen et al. [32], we used questionnaire data from baseline, 6, 12, 18, and 24 month by taking the measurement of the closest visit or the average of the closest two visits in our analysis. More than 75% (N=254) of families have complete data for all five visits. Multivariate imputation [33] was applied to impute missing values. All families received assistance with appointment scheduling; those in the intervention arm additionally participated in sessions with a specially trained health advisor at each clinic visit. Intervention activities included preparation assistance by phone before each clinic visit, the “WE-CAN” manage diabetes applied problem-solving approach during the visit, and follow-up phone consultation after the visit. Self-report questionnaires were completed at each study visit.

The parent and the child separately completed identical questionnaires for assessment of parent task involvement (DFR) and Parent–Child conflict (PCC). These give rise to four manifesto scores: child-reported DFR (CDFR), parent-reported DFR (PDFR), child-reported PCC (CPCC), and parent-reported PCC (PPCC), which we use to infer the underlying Parent–Child relationship in this paper. Figure A.1 in Supplementary Material provides violin plots of these outcomes over time stratified by treatment arms. In general, children reported lower parent task involvement and slightly higher Parent–Child conflict scores than parents. Moreover, parent task involvement declined and Parent–Child conflict remained relatively stable over time.

Early preliminary analyses of FMOD trial data have applied the latent class model (LCM, i.e., finite Gaussian mixture model) [34], separately to baseline and 24-month data with the goal to estimate underlying Parent–Child relations at the start and the end of the study, and found that an LCM with 3 latent classes fit the data well at both times [32]. The estimated Parent–Child relationship classes consist of a Discordant class, characterized by high degree of parent involvement and high parent–child conflict, a Harmonious class, characterized by high parent involvement and low conflict, and an Indifferent class, characterized by low parent involvement and low conflict; see Table 1.

While providing a rough picture of the Parent–Child relationship at the start and end of the study, these preliminary analyses treated data from baseline and 24 month independently and ignored the longitudinal feature of the FMOD trial data.

To understand the dynamics of unobserved Parent–Child relations, a natural extension of the above LCM approach is the hidden Markov model. To that end, we fit a multivariate mixed effect HMM (MHMM) [20] to the FMOD trial data, without differentiating between child and parent (hereafter we call it “the Family model”). In MHMM, the conditional distribution of each outcome given the current hidden state, or so-called emission probability model, is specified as a Gaussian distribution with mean resulting from both the subject-specific random effect and the fixed effect of the underlying hidden state, and variance resulting from the fixed effect of the underlying hidden state. We applied the widely applicable information criterion (WAIC) [35] for model selection. It suggests that a 3-state HMM fits the data well. In Supplementary Material, the top part of Table A.1 reports estimated fixed effects of the three hidden states and the top part of Table A.2 provides estimated initial and transition probabilities by treatment arms.

We first note that the pattern in estimated fixed effects of each state from the Family model closely resembles that displayed in Table 1, suggesting that the same three latent classes of Parent–Child relationship, namely Discordant, Harmonious, and Indifferent, are underlying the manifesto variables. At baseline, about 43% and 49% families are in Discordant and Harmonious states, respectively, with the remaining in the Indifferent state (Table A.2). The estimated transition matrices in Table A.2 indicate that families are more likely to move away from the Discordant state and to stay in the Harmonious state when they are in the intervention versus the usual-care arm. In order to further test the treatment differences, we obtain the 95% posterior credible intervals of the average transition probability differences and average stationary probability differences [24] and display them in Fig. A.2 in Supplementary Material. Despite not significant, the differences show that families in the intervention arm are less likely to stay in the Discordant state and more likely to move into the Harmonious state. According to the stationary probability differences, families in the intervention arm are more likely to transition to the Harmonious state at the end of the study.

A limitation of this application of HMM to the FMOD trial data is that it assumes the same Markov process between child and parent. Since the manifesto variables were obtained from the parent and the child separately, it is possible that they reflect different perceptions. Understanding these differential perceptions is important as it can shed insights in how the intervention works.

As a naive way to gauge these differences, we repeated the above MHMM to parent- and child-specific outcomes separately (hereafter call them the “Parent model” and the “Child model”, respectively) and ended up with a three-state model for each. These results are reported in the second and third parts of Table A.1 for emission model parameters and of Table A.2 for initial and transition probabilities, respectively. Several observations can be made. First, as in LCM and the Family model, the estimated latent classes/states in both Parent and Child models agree with those in Table 1. That is, the three identified states are featured by high Parent–Child conflict and high parent task involvement, low conflict and high involvement, and low conflict and low involvement, respectively. Although states from different models are centered at different values, we name them in the same way as the “Family model.” The within-dyad similarity stops here, however. While 35%, 44%, and 21% families at baseline are in Discordant, Harmonious, and Indifferent states according to the Child model, the corresponding prevalence according to the Parent model are 18%, 76%, and 6%, respectively (Table A.2). Transition probabilities also differ substantially between the parent and the child. For example, while 35% in the usual-care arm and 23% in the intervention arm stay in the Discordant state by the Child model, 77% and 71% do the same according to the Parent model. These differences in transition probabilities are evident by noticing the low diagonal entries (0.24, 0.53, and 0.52) in Table A.3 which cross-tabulates estimated classes at each visit from the Parent and Child models, respectively.

It is clear that there is a significant heterogeneity between child’s and parent’s perceptions of Parent–Child relations. The application of an HMM using all data (the Family model) fails to account for this heterogeneity. On the other hand, parent- and child-specific HMMs result in information loss and render results challenging to interpret. The next section will describe the proposed approach that aims at bridging this gap and provides a mechanism to address the research question of differential perceptions from the parent and the child toward Parent–Child relations.

## 3 The Perception-Augmented HMM (pHMM)

### 3.1 Overview

**Fig. 1:**
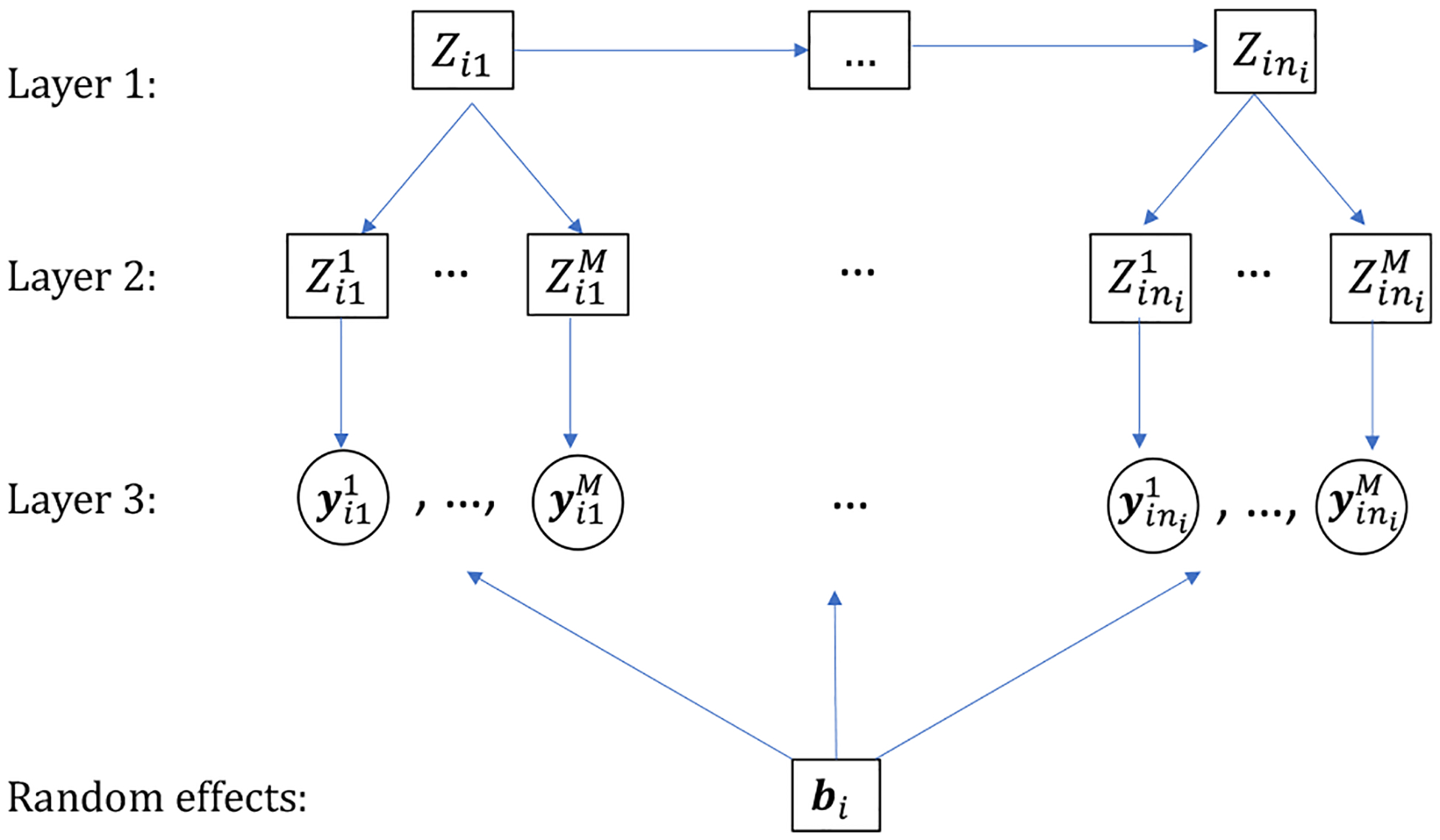
Schematic overview of the perception-augmented hidden Markov model structure for the ith cluster, with cluster-level hidden state process ${\text{Z}}_{i}$, member-level hidden state process ${\text{Z}}^{\text{m}}$, observed process ${\text{y}}_{i}$, and random effects ${\text{b}}_{\text{i}}$. In the diagram, n is the number of follow-ups, and M is the number of members in one cluster

The proposed new hidden Markov model is intended for longitudinal multivariate data from members of a cluster where cluster-level states can be differentially perceived by each member. For ease of understanding, the methodological development below will be in the context of the FMOD trial where family is the cluster and child and parent are the corresponding cluster members. We will also use class interchangeably with state when describing the hidden Parent–Child relationship, and sometimes omit the word “latent” if no confusion arises. The proposed framework consists of three layers of dependent processes, as illustrated schematically in Fig. 1. The first layer is a Markov process of the family-level states that are assumed to adhere to the first-order Markov assumption with initial and transition probabilities. The second layer comprises the hidden member-level states conditional on the first layer. In this layer, child and parent interpret the family-level state and form their own states. We call this interpreting process the “perceiving” process and the associated matrix of probabilities the “perception matrix”. We assume that the perception matrix is homogeneous across time but can be different for family members and treatment arms. The third layer is the emission process. It specifies the mechanism by which the outcomes are manifested through the member-level states. Given the current family-level states, member-level states are assumed to be independent of each other. Similarly, given the member-level states, current observed variables are independent of past and future ones.

### 3.2 The Family-Level Markov Process

Let $Z_{it}$ denote the unobserved family-level Parent–Child relationship state of family i at time $t,i=1,\ldots ,N,t=1,\ldots ,n_{i}$, where N is the number of families in the study and $n_{i}$ is the number of follow-ups of family i. Further, denote ${\text{Z}}_{i}=\left(Z_{i1},\ldots ,Z_{in_{i}}\right)$ to be the state sequence of family i and Z to be the collection of all ${\text{Z}}_{i}$’s. Let $s_{i}\in \{1,2,\ldots ,S\}$ represent the treatment arm assigned to family i, where S is the total number of arms. In FMOD trial, S=2 as the study treatments consist of the usual-care and intervention arms. Since our primary interest is in whether study treatment affects family transitioning from one Parent–Child relationship state to another, we allow for separate transition and initial probabilities.

Specifically, we assume that ${\text{Z}}_{i}$ arises from a Markov process of state space {1,…,C} with transition matrix ${\text{P}}^{s_{i}}=\left\{P_{kj}^{s_{i}}\right\}$ and initial probability vector $\pi^{s_{i}}=\left\{\pi_{j}^{s_{i}}\right\}$, where C is the number of latent Parent–Child relationship states, j,k=1,…,C, and $s_{i}=1,\ldots ,S$. Given the first-order property and time homogeneity, we can write the Markov chain of the hidden states ${\text{Z}}_{i}$ as follows:

$$P\left(Z_{i1}=j|s_{i}=s\right)=\pi_{j}^{s},\;P\left(Z_{it}=j|Z_{i,t-1}=k,s_{i}=s\right)=P_{kj}^{s},\;t=2,\ldots ,n_{i}.$$

We note that other alternative formulations of the treatment effects on transition and initial probabilities are possible [24]. One straightforward extension is to incorporate in-homogeneous hidden process [36], which allows time-varying treatment effects, by estimating different transition matrix for each observational period. The primary advantage of our formulation is that it leads to simpler computational algorithm.

### 3.3 The Perception Process

It is possible that the family-level Parent–Child relationship state can be differentially perceived by child and parent. Theoretical work in developmental psychology has long recognized this [21–23]. Empirically, we see from Fig. A.1 that there is a discernible difference in the two manifesto scores between parents and children. In particular, compared to parents, children tend to give lower scores in parent task involvement (DFR) (33.05 vs. 35.51), suggesting that they perceive less task involvement from parents. Similarly, children tend to give slightly higher scores in Parent–Child conflict (PCC). As such, it is helpful to allow this differential perception in the modeling framework. To that end, for member m∈{1,…,M} in family i at time point t, let $Z_{it}^{m}\in \left\{1,\ldots ,C^{m}\right\}$ denote the member-specific hidden state where M is the number of members in a cluster and $C^{m}$ is the member-specific number of states. In FMOD trial, M=2. To model the perception process, we assume that $Z_{it}^{m}$ are mutually independent across m, for fixed i and t given the current state $Z_{it}$ and that

$$P\left(Z_{it}^{m}=j|Z_{it}=k,s_{i}=s\right)=Q_{mkj}^{s},\;t=1,\ldots ,n_{i},$$

where $Q_{mkj}^{s}$ is the probability that the cluster-level state k is perceived as j by member m in study arm s. To simplify notations and focus on the main idea, we will assume member-specific but study-arm-common perception matrices in the following development so that superscript s is dropped in perception probability $Q_{mkj}^{s}$. Let ${\text{Q}}_{m}$ be the collection of $Q_{mkj}$’s.

### 3.4 The Emission Model

Let ${\text{y}}_{it}=\left({\text{y}}_{it}^{1},\ldots ,{\text{y}}_{it}^{M}\right)$ denote a multivariate longitudinal response for cluster i at time t. Specifically, ${\text{y}}_{it}^{m}=\left(y_{i1t}^{m},\ldots ,y_{iJ_{m}t}^{m}\right)$ is a $J_{m}$-dimensional outcome from member m in cluster i at time t. Here $J_{m}$ is the number of outcomes associated with member m. In the FMOD trail, $J_{m}=2$ for all m. Random vectors ${\text{y}}_{it}^{m}$ and ${\text{y}}_{it^{\prime }}^{m}$ are independent given the current member states $Z_{it}^{m}$ and $Z_{it^{\prime }}^{m}$, for $t\neq t^{\prime }$. In addition, to account for heterogeneity introduced by individual clusters, we assume *i.i.d* cluster-specific random effects ${\text{b}}_{i}=\left(b_{i1},\ldots ,b_{ir}\right)$ with a common distribution $f\left({\text{b}}_{i}|\Sigma \right)$, e.g., zero-mean multivariate normal, where $r=\sum_{m}J_{m}$ and Σ is an unknown covariance matrix, which characterizes the correlation pattern of the longitudinal observable variables.

Given member’s hidden states $Z_{it}^{m}$ and the random effect ${\text{b}}_{i}$ of cluster i, the jth response outcome $y_{ijt}^{m}$ from member m in cluster i follows a normal distribution:

(1) $$y_{ijt}^{m}|Z_{it}^{m}=k,{\text{b}}_{i},\tau_{j}^{m},\beta_{j}^{m},\gamma_{j}^{m}\sim N\left({\text{u}}_{itk}^{T}\tau_{j}^{m}+{\text{X}}_{it}^{T}\beta_{j}^{m}+\omega_{ij}^{T}{\text{b}}_{i},{\text{u}}_{itk}^{T}\gamma_{j}^{m}\right),$$

where ${\text{u}}_{itk}$ is a $C^{m}$-dimensional vector defining the contrast between latent states, with first k elements 1 and others 0, $\tau_{j}^{m}=\left(\tau_{j,1}^{m},\ldots ,\tau_{j,C^{m}}^{m}\right)$ represents the fixed effect intercept for all hidden states, ${\text{X}}_{it}=\left(X_{it1},\ldots ,X_{itq}\right)$ are the covariates of cluster i at time $t,\beta_{j}^{m}=\left(\beta_{j1}^{m},\ldots ,\beta_{jq}^{m}\right)$ are the associated coefficient vector, $\omega_{ij}$ is a r-dimensional vector, and $\gamma_{j}^{m}=\left(\gamma_{j,1}^{m},\ldots ,\gamma_{j,C^{m}}^{m}\right)$ the fixed effect on variance of the jth outcome variable from member m for the hidden states. We follow the same parameterization as in Scott [37] and in Raffa and Dubin [20]: The term ${\text{u}}_{itk}^{T}\tau_{j}^{m}$ gives the fixed effect intercept of State k as the cumulative sum of the first k elements in $\tau_{j}^{m}$. Similarly, $u_{itk}^{T}\gamma_{j}^{m}$ gives the variance of the kth state as the cumulative sum of the first k elements in $\gamma_{j}^{m}$. The vector $\omega_{ij}$ is used to express the random effect of variable j of cluster i, with value 1 at jth place of the vector and 0’s elsewhere. In this way, we define separate but correlated random effects of all response variables from cluster i.

## 4 Estimations

The above specifications are tailored for the FMOD data and lead to a straightforward Bayesian computational algorithm. They can be easily generalized to more complex situations and to incorporate responses from other distributions. The marginal likelihood function given the above model formulations is

(2) $$L\left(\Theta ;\text{y}\right)=\prod_{i=1}^{N}\int_{{\text{b}}_{i}}\alpha_{in_{i}}1f_{{\text{b}}_{i}}\left({\text{b}}_{i}|\Sigma \right)\text{d}{\text{b}}_{i},$$

where y is the collection of all ${\text{y}}_{it},\Theta$ consists of all parameters, including those associated with the Markov chains (${\text{P}}^{s},\pi^{s}$, and ${\text{Q}}_{m}^{s}$), with the emission probability ($\beta =\left\{\beta_{j}^{m}\right\},\tau =\left\{\tau_{j}^{m}\right\}$, and $\gamma =\left\{\gamma_{j}^{m}\right\}$), and those with the random effect (Σ), $\alpha_{in_{i}}$ is the forward probability for the ith cluster at time $n_{i}$, and 1 is a vector of 1’s. More specifically, $\alpha_{i1}=\pi^{s}G\left({\text{y}}_{i1}\right)$ for t=1, and $\alpha_{i,t}=\alpha_{i,t-1}P^{s_{i}}\text{G}\left({\text{y}}_{it}\right)$ for $t=2,\ldots ,n_{i}$, where $G\left({\text{y}}_{it}\right)$ is a diagonal matrix with entries $\prod_{m=1}^{M}\prod_{j=1}^{J_{m}}\sum_{k=1}^{C^{m}}f\left(y_{ijt}^{m}|Z_{it}^{m}=k,{\text{b}}_{i},\Theta \right)*Q_{mkh},h=1,\ldots ,C$.

In order to deal with multiple members, each providing multiple response variables in the clusters, we need a flexible way for estimation with reasonable computational efficiency. With the model specifications introduced in Sect. 3, we propose a Gibbs sampler to fit the model. This also eliminates the challenges in evaluating the high dimensional integrals related to the random effects in the model. Further, the Bayesian framework is straightforward to incorporate more complicated specifications.

We specify independent conjugate priors for components of parameter vector Θ as follows:

(3) $$\Theta =\left[\beta ,\tau ,\gamma ,{\text{Q}}_{m},\text{P},\pi ,\Sigma \right]\;=[\beta ,\tau ]\times \left[\gamma \right]\times \left[{\text{Q}}_{m}\right]\times \left[\text{P}\right]\times \left[\pi \right]\times \left[\Sigma \right]\;=1\times \prod_{m=1}^{M}\prod_{j=1}^{J_{m}}IG\left(a,b\right)\times \prod_{m=1}^{M}\prod_{k=1}^{C}Dir\left(1_{C^{m}}\right)\times \prod_{k=1}^{C}Dir\left(1_{C}\right)\times Dir\left(1_{C}\right)\times IW\left(\sum_{m=1}^{M}J_{m}+1,\text{I}\right),$$

where I is an identity matrix with conforming dimension, IG, Dir and IW represent inverse Gamma, Dirichlet and inverse Wishart distributions, respectively. Based on these prior specifications, we can obtain a Gibbs sampler to sample from posterior [Θ,b∣y] where b is the collection of all ${\text{b}}_{i}$’s. Specifically, the sampling procedure alternates between sampling from $\left[\text{Z},{\text{Z}}_{m}|\text{y},\Theta ,\text{b}\right]$ and $\left[\Theta ,\text{b}|\text{Z},{\text{Z}}_{m},\text{y}\right]$. We use stochastic recursion to sample from [Z∣y,Θ,b], with the hidden states of clusters sampled separately by calculating the forward probabilities $\alpha_{i}$. The hidden state of each member is then sampled accordingly with the current perception matrix. Once the samples from $\left[\text{Z},{\text{Z}}_{m}|\text{y},\Theta ,\text{b}\right]$ are updated, sampling from $\left[\Theta ,\text{b}|\text{Z},{\text{Z}}_{m},\text{y}\right]$ is straightforward.

## 5 Revisit of the FMOD Trial Data

We re-analyze the FMOD trial data with the proposed perception-augmented HMM framework. Its emission component is specified as in (1) with $N=390,n_{i}\equiv n=5,M=2$, and $J_{m}\equiv J=2$. We adjusted for age at baseline and gender of youth in the emission model so that $X_{it}=({\text{age}}_{i},{\text{gender}}_{i})$. About 49% of youth in the study cohort are male and the average age at baseline is about 12.5 years. The study treatment (s=1,2 for usual care and intervention, respectively) was used in modeling transition probabilities so that arm-specific transition matrices are estimated.

### 5.1 Number of Latent Parent–Child Relationship States

As with any HMM, a key model selection component in the proposed pHMM is the number of latent Parent–Child relationship states. While pHMM accommodates varying numbers of latent states between family, parent, and child, we consider the case where a common number is assumed. We use the widely applicable information criterion (WAIC Watanabe [35]). A noted feature of WAIC and related information criteria is that they generally decreases as the number of latent states increases [20]. We observed similar phenomenon in our simulation (see Sect. 6.2 and Table C.1). As a result, we balance WAIC and model parsimony to select the number of latent states. For the FMOD trial data, WAIC values of pHMM with 2, 3, and 4 common latent Parent–Child relationship states are 14,963.8, 13,577.2, and 12,926.8, respectively. Although the model with four states has the lowest WAIC value, the reduction is the biggest from 2 to 3 states. Moreover, two out of the four states are featured as the “Discordant” class in Table 1. Combining this observation with results in LCM and standard MHMM analyses, and for ease of interpretation, we decide to base on the model with three common latent states (hereafter the 3-state pHMM) to report our primary results. A sensitivity analysis (see Table B.1) allowing different numbers of latent states between family, parent, and child corroborate the above findings that the 3-state model has the largest improvement compared to those with fewer states and smaller improvement compared with those with more states.

### 5.2 Model Parameter Estimates

**Fig. 2:**
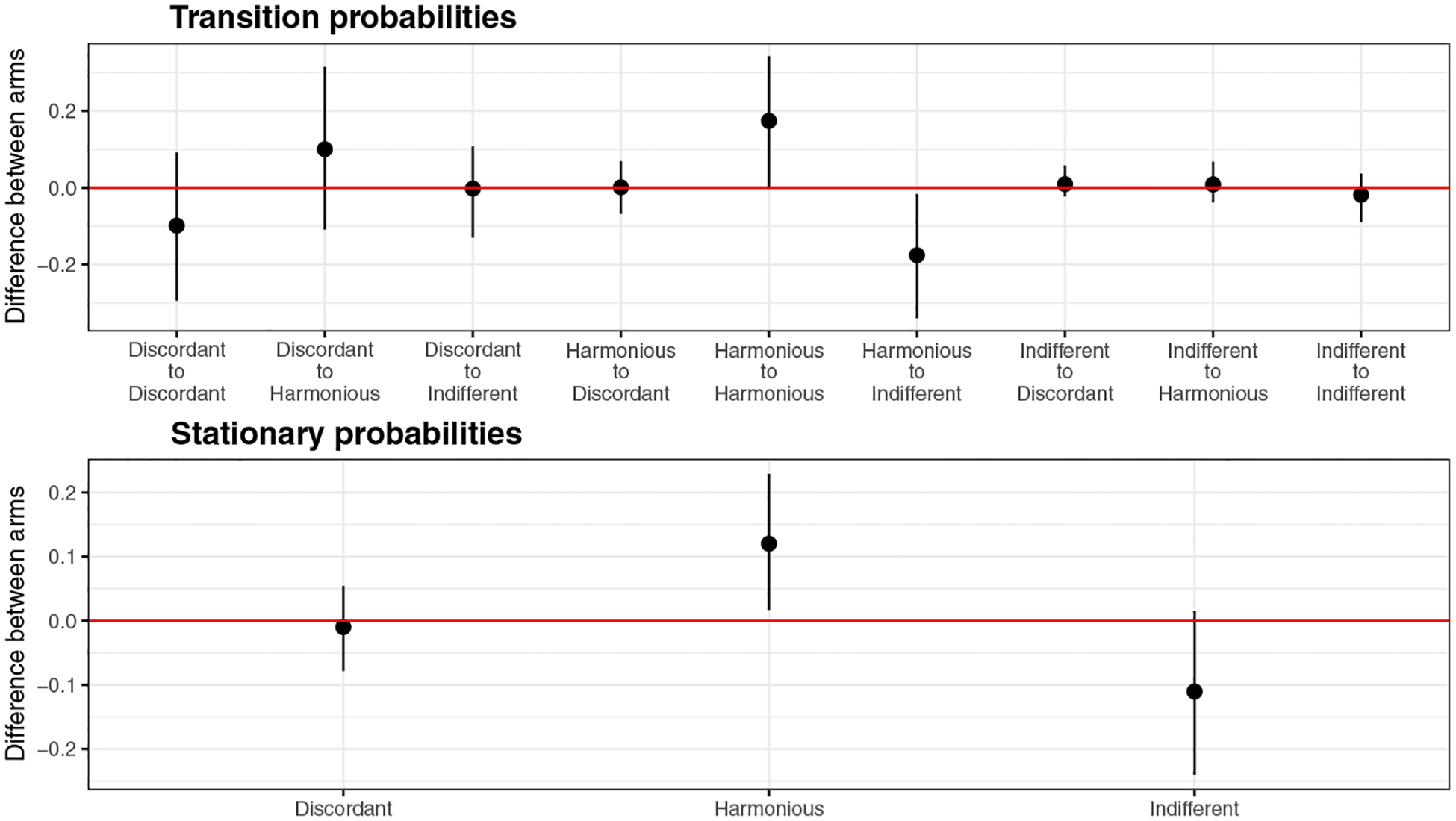
95% posterior credible intervals of the average transition probability differences and average stationary probability differences between intervention and usual-care arms over all families from the 3-state pHMM

Basing on the 3-state pHMM, we report estimated emission model parameters in Table 2, initial and transition probabilities in Table 3, and perception matrices in Table 4, respectively. We first note that, according to emission model parameter estimates, the three latent states identified by the 3-state pHMM have the same characteristics as those in Table 1, suggesting that the same three classes of relationship can be used here. From Table 3, we see that, at baseline, about half of families are in Discordant class, and 44% and 6% in Harmonious and Indifferent classes, respectively. While families in the Indifferent class show little difference in transition probabilities between intervention and usual-care arms [(0.01, 0.01, 0.98) versus (0.02, 0.02, 0.96)], families in the other two classes show discernible differential transition behavior. More specifically, intervention families are more likely to move out of the Discordant class (0.52, 95% CI 0.34–0.71) than usual-care families (0.42, 0.25–0.59), although not statistically significant. These families mostly move into the Harmonious class, slightly more so for those in intervention than in usual-care. Moreover, families are more likely to stay in the Harmonious class when in the intervention arm (0.65, 0.51–0.75) than in the usual-care arm (0.47, 0.30–0.61), and less likely to stay in the Indifferent class when in intervention (0.96, 0.89–0.99) than in usual-care (0.98, 0.93–1.00). These findings are further tested by comparing the average transition probabilities and stationary probabilities between the intervention arm and the usual-care arm as shown in Fig. 2. From Fig. 2, we see some of the trends are statistically significant. For example, intervention arm has a significantly higher probability to transit into harmonious state in the end.

**Fig. 3:**
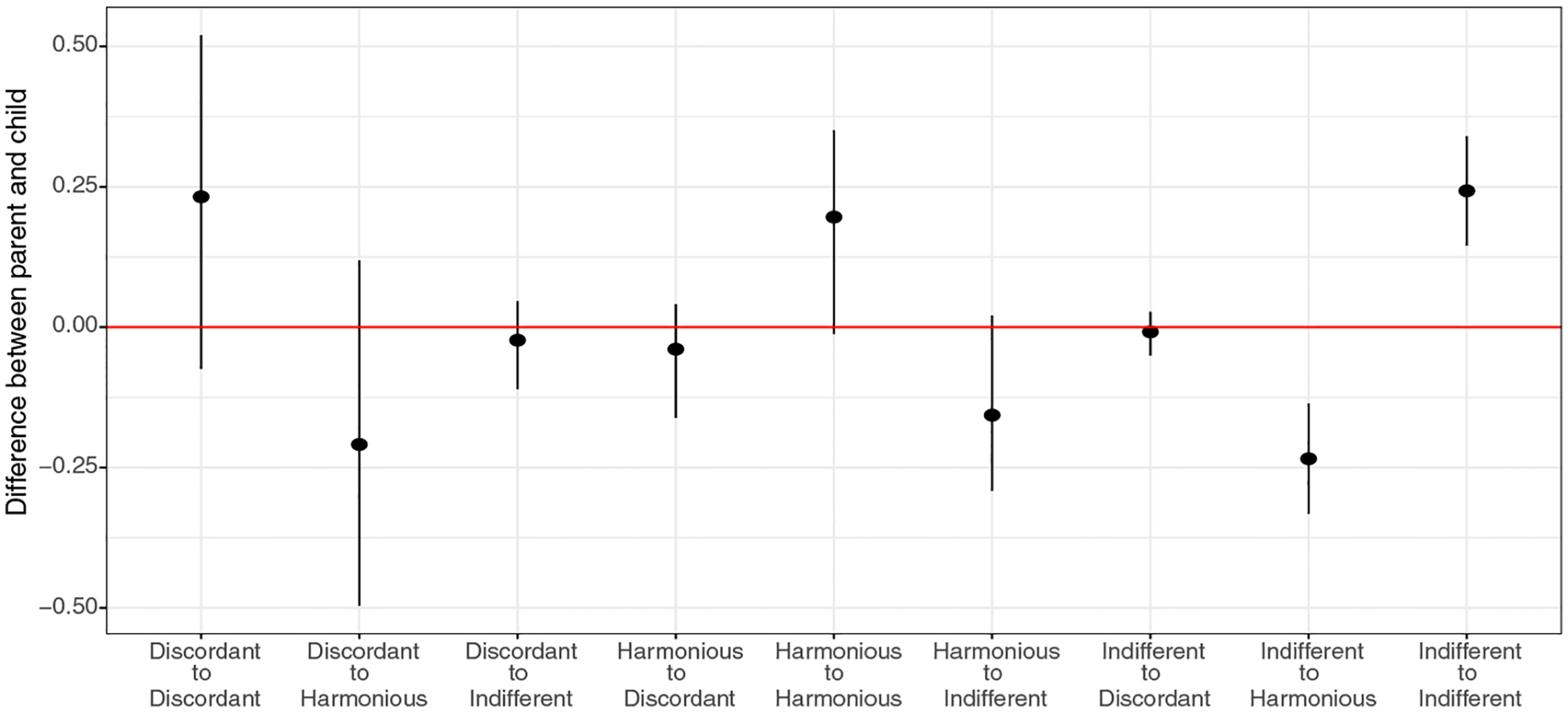
95% posterior credible intervals of the perception probability differences between the parent and the child from the 3-state pHMM

Turning to perception probabilities in Table 4, we first note that child tends to have a more diverging view toward family-level Parent–Child relation than parent—this is reflected in the smaller diagonal entries in the perception matrix of children than that of parents. For example, while a Discordant Parent–Child relationship is perceived by child to be discordant only 52% of the time, it is perceived so by parent 75% of the time. Figure 3 displays 95% posterior interval for average differences between parent’s and child’s perception matrices. It shows higher diagonal entries (Discordant–Discordant, Harmonious–Harmonious, Indifferent–Indifferent) of parent perception matrix, indicating that parents are more likely to perceive the Parent–Child relationship as it is than children.

We note that the 3-state pHMM (WAIC = 13,577.2) fits the FMOD trial data better than the Family model with three states in Sect. 2 (WAIC = 14,528.2), and also better than an extended pHMM where treatment-specific perception matrices are allowed (WAIC = 16,862.2). The latter result suggests that perception probabilities of child and parent are not associated with study arms.

### 5.3 Model Goodness of Fit and Diagnostics

Although the 3-state pHMM is chosen as the best model, it is important to investigate whether it fits the data well. In this section, we check the goodness of fit of the proposed method using posterior predictive checks [38]. The idea is to examine whether observed statistics are close to their predicted counterparts from the model. If the model is a good fit of the data, the observed statistics should not be in extreme quantiles of the predictive distributions. As we assumed that the four outcomes are normally distributed (after logarithmic transformation in PCC), we conduct these goodness of fit checks using their means and variances. This gives rise to eight observed statistics (four means and four variances). To obtain the posterior predictive distributions, we utilize the MCMC samples of the model parameters and simulated 1000 sets of outcomes. We then construct the posterior distributions of the eight statistics and compare the observed values of the statistics with their corresponding simulated distributions (Table 5). We find that the observed means and variances are not located in the extreme tails of the respective distributions, with their placements (quantiles) ranging from 8 to 53%. These suggest that there is no evidence of lack-of-fit issue when applying the 3-state pHMM to the FMOD trial data. The QQ-plots of pseudo-residuals [39] in Fig. B.1 in Supplementary Material also show adequate predictive performance.

### 5.4 Computational Considerations

In the primary analysis of FMOD trial data using the 3-state pHMM, we used the following prior specifications:

$$\pi \sim Dir\left(1_{C}\right),\;P_{k}^{s_{i}}\sim Dir\left(1_{C}\right),\;k=1,\ldots ,C\;\Sigma \sim IW\left(\sum_{m=1}^{M}J_{m}+1,\text{I}\right),\;{\text{Q}}_{m,k}^{s_{i}}\sim Dir\left(1_{C^{m}}\right),\;k=1,\ldots ,C,\;\gamma_{lk}^{2,m}\sim IG(0.001,0.0002),\;k=1,\ldots ,C_{m},\;l=1,2,$$

where $C^{m}=C=3,M=2$, and $J_{m}=2$. Moreover, we assume that regression coefficient vectors τ and β take non-informative priors. In our MCMC implementation, we first ran 4000 iterations with 3000 as burn-in and used the mean of converged value as the initial values for the second round. In the second round, we ran 40,000 iterations with 30,000 as burn-in. Trace plots and diagnostic tools were used to ensure convergence of the algorithm. Trace plots of selected model parameters are presented in Fig. B.2. We noticed no label switching in the analysis of FMOD trial data.

## 6 Simulation Studies

To evaluate the performance of the proposed model, we conduct three simulation studies under different scenarios. Section 6.1 introduces simulation setup, Sect. 6.2 assesses model selection performance of WAIC, Section 6.3 evaluates parameter estimations, and Sect. 6.4 examines latent class estimation.

### 6.1 Simulation Setup

Since we are comparing pHMM to both MHMM and an extended pHMM where treatment-specific perception matrices are allowed (hereafter pHMMe), we create three scenarios by using each of them as the true model to generate data, and then fit all three models under each scenario. To mimic the real data application, we set the true parameters at the estimated values obtained in Sects. 2 and 5 and assume that the true number of hidden states is 3 for all three scenarios. As in FMOD trial, 390 families are included in each dataset, with each family followed up five times.

The steps of generating the simulation data are as follows. First, we generate the hidden states of the family as a Markov chain and the random effects following multivariate normal distribution independently. Second, when MHMM is the true model, we generate the observed process under MHMM framework. When pHMM is the true model, we generate the hidden states of the members with the member- and/or treatment arm-specific perception matrices. Third, with the members’ hidden states, we generated the observed process. For each scenario, we constructed 1000 random replications and fit MHMM, pHMM, and pHMMe to each.

### 6.2 On Model Selection Using WAIC

To evaluate the performance of WAIC in selecting number of latent states, we fit 2-, 3-, 4-, and 5-state pHMMs to the data generated by the 3-state pHMM. Table C.1 contains results based on 1000 simulation runs. We see that WAIC prefers more complicated model than simpler ones. There is a drop of about 1600 units in WAIC from the 2-state model to the 3-state model. However, the reduction in WAIC from 3- to 4-state models is much smaller (at around 500).

### 6.3 On Parameter Estimation

In this part, we will focus on assessing performances of the three models in estimating initial probabilities, transition probabilities and perception matrices under each scenario by evaluating bias and coverage probability of 95% credible interval based on 1000 random simulations.

#### 6.3.1 Scenario 1: MHMM as True Model

When data are generated from an MHMM, both pHMM and pHMMe perform well in estimating the initial probabilities and the transition matrices (Table C.2). The posterior means are all close to the true values with reasonably small variations. The 95% credible intervals contain the true values at close to the nominal level.

The true perception matrix under this scenario is the identity matrix. As a fitting model, MHMM does not estimate perception matrices directly. However, we can approximate the perception matrices by estimating the hidden states of the trios of family, parent, and child separately and cross-tabulating them. There are two ways to arrive at these hidden state estimates. One approach is to estimate them independently for parent, child and the whole family using the corresponding data. We call this approach “unconstrained” hereafter. The other approach is to estimate the members’ states while fixing the emission probability model parameters at those obtained from the Family model. We call this approach “constrained” hereafter. Table C.3 reports estimated perception matrices. Since the approximations under MHMM are ad hoc, no posterior standard deviations are provided. Instead, we report the sample standard deviation of the posterior means over all simulation runs to reflect the variability of the estimation. The coverage probability is not applicable here either, as the true perception matrices have values that are at boundaries of unit interval. We observe that, under MHMM, the estimated perception matrices for parent are different from the identity matrix under both constrained and unconstrained approaches. However, both pHMM and pHMMe have unbiased estimates of the perception matrices. In particular, when assuming two sets of perception matrices for the two study arms under pHMMe, both sets of perception matrices are close to each other and to the identity matrix.

#### 6.3.2 Scenario 2: pHMM as True Model

When generating data with pHMM, we assume different perception matrices for child and parent and set the true values at the estimated perception matrices in Sect. 5. In this case, child tends to perceive hidden State 1 as State 2 more likely than parent. From the estimation results of initial probabilities and transition matrices in Table C.4, we see that while pHMM and pHMMe both produce unbiased and reasonably precise estimates, MHMM results in very biased estimates of initial probabilities. While the true values are 0.76 and 0.18 for States 1 and 2, respectively, the corresponding estimates from MHMM are 0.13 and 0.78. Estimates of some transition probabilities are also biased. For example the probability transiting from State 2 to 1 for the usual-care arm has a 95% CI (0.066, 0.213) which does not contain the true value of 0.029. The perception matrices were estimated well by both pHMM and pHMMe (Table C.5). As expected, pHMMe returned similar perception matrices for both study arms. Under MHMM, however, both approaches to approximate perception matrices performed poorly, with the estimated perception matrices similar to each other but very different from the truth. This poor performance again confirms that MHMM is not capable of addressing differential perceptions adequately.

#### 6.3.3 Scenario 3: pHMMe as True Model

In this scenario, we assume that both the child and parent perception matrices are study arm specific, so that the intervention plays a role in changing both transition and perception patterns. Generally, the simulation results are similar to Scenario 2. For initial probability and transition matrices (Table C.6), both pHMM and pHMMe return unbiased estimates. On the other hand, MHMM return biased initial probabilities and probabilities transiting between the first two states. For perception matrices (Table C.7), pHMM returns estimates approximately as the average of the two true perception matrices for the two study arms. The estimates from MHMM are either far from the true value or have too much variation, especially with the unconstrained approach.

### 6.4 On Latent State Estimation

Performance in calling latent states is another very important assessment of interest. We evaluate pHMM and pHMMe of this aspect through several popular classification criteria [40] under the three scenarios. For comparison, we also evaluated MHMM using the same set of criteria. Let $tp_{z},tn_{z},fp_{z}$ and $fn_{z}$ stand for true positive, true negative, false positive and false negative respectively for the zth class, z=1,…,C, respectively. The set of criteria we used are as follows:

- Accuracy: $\sum_{z=1}^{C}\frac{tp_{z}+tn_{z}}{tp_{z}+tn_{z}+fp_{z}+fn_{z}}/C$, which is the average per-class effectiveness of a classifier;
- Error Rate: $\sum_{z=1}^{C}\frac{fp_{z}+fn_{z}}{tp_{z}+tn_{z}+fp_{z}+fn_{z}}/C$, which is the average per-class classification error;
- Micro-precision $(\left.{\text{Precision}}_{m}\right):\sum_{z=1}^{C}tp_{z}/\sum_{z=1}^{C}\left(tp_{z}+fp_{z}\right)$, which indicates the agreement of the true class with the estimated class. This micro version of precision is equal to micro-recall and micro F-score;
- Precision: $\sum_{z=1}^{C}\frac{tp_{z}}{tp_{z}+fp_{z}}/C$, which is an average per-class agreement of the true class with the estimated labels;
- Recall: $\sum_{z=1}^{C}\frac{tp_{z}}{tp_{z}+fn_{z}}/C$, which is an average per-class effectiveness to identify each class;
- *F*-score: {Precision × Recall}/{Precision + Recall}, which indicates the relation between the true positive labels and the estimated positive labels bases on a per-class average.

From Table C.8, we see that the true model generally has the best performance in identifying latent states. In the first scenario, where data are generated from MHMM, MHMM has the best performance in calling states with respect to all metrics. At the same time, we observe that the performance of pHMM and pHMMe are reasonable, too. In the second scenario, pHMM has the best performance in calling latent state of family as well as of child’s and parent’s perceptions. While pHMMe has relatively good performance in comparison, MHMM performs much worse in identifying the correct latent states. When pHMMe is the true model, we observe good results from both pHMM and pHMMe. Still, MHMM returns poor latent state calling performance.

## 7 Conclusions

Optimal glycemic control in youth with T1D requires effective Parent–Child teamwork. Thus, the mechanism for a behavioral intervention to reduce deterioration in glycemic control may be through its effect on improved Parent–Child relationship. We have proposed a novel modeling framework that allows us to test whether the intervention improves Parent–Child relationship while at the same time accommodating heterogeneity in perceptions that the parent and the child have toward the underlying family relation. This framework extends standard HMM by adding a perception layer and enjoys many nice features of HMM in structured and efficient estimation algorithm. Based on this, it is of interest to develop mediation analysis tools in the future to test the intervention’s effect on glycemic control through parent–child relationship.

The substantive findings from the FMOD trial data are interesting. Families in the intervention arm were more likely to stay in the Harmonious Parent–Child relation state and less likely to move from Harmonious to Indifferent. Intervention families were also less likely to stay in Discordant and more likely to move from Discordant to Harmonious state. Although there was no evidence of different perceptions in different treatment arms, they varied between children and parents. In general, parents were more likely to perceive the Parent–Child relationship as it is than were children.

Given the large number of parameters in the model, the moderate sample size of the FMOD trial data can potentially limit the statistical power of the study. A study with more families could mitigate this problem while also serving to validate the findings. For simplicity, we made several assumptions, including time-homogeneous, first-order hidden Markov process, conditionally independent perception processes, and adjusted covariates only in emission models. These assumptions pose several limitations which can be addressed in future works. First, Scott et al. [36] have proposed an in-homogeneous but common hidden process, which allows different transition matrices from the same distribution at different observational period. We believe it could be adapted to the model proposed here. Second, in FMOD study, we collected data from the same two types of measurements from both parent and child, which are correlated in nature. We accounted for these correlations in random effects only. Some alternative ways are worthwhile investigating in the future work, including dependent perception processes. Last, the proposed model adjusted for covariates only in the emission probability model. Future work could explore different strategies to accommodate covariates in transition and/or perception matrices.

Due to its singularity, determining the number of hidden states is always a difficult task for HMM. There are only a few techniques that we can use for model selection. We applied WAIC for the real application and tested its performance in simulation studies. It generally prefers higher number of hidden states. The explanation of this phenomenon and best practices of using it merit separate investigations in future works.

## Supplementary Material

Supplementary Material

**Supplementary Information** The online version contains supplementary material available at https://doi.org/10.1007/s12561-022-09360-8.

## Figures and Tables

**Table 1:** Characteristics of estimated Parent–Child relation classes from latent class model applied to the FMOD trial data

Outcome	Parent–child relation class		
	Discordant	Harmonious	Indifferent
Parent task involvement (DFR)			
Child-reported DFR (CDFR)	High	High	Low
Parent-reported DFR (PDFR)	High	High	Low
Parent-child conflict (PCC)			
Child-reported PCC (CPCC)	High	Low	Low
Parent-reported PCC (PPCC)	High	Low	Low

**Table 2:** Posterior means and 95% credible intervals of fixed effects in the 3-state pHMM for the FMOD trial data

Outcome	Parent-child relation class		
	Discordant	Harmonious	Indifferent
CDFR	33.90 (32.75, 35.01)	34.29 (33.51, 35.09)	30.63 (29.83, 31.47)
CPCC	3.33 (3.26, 3.40)	3.19 (3.14, 3.23)	3.09 (3.04, 3.14)
PDFR	38.87 (37.84, 39.87)	36.93 (36.03, 37.74)	33.75 (32.88, 34.59)
PPCC	3.32 (3.26, 3.39)	3.22 (3.18, 3.26)	3.23 (3.19, 3.26)

CDFR and PDFR are child- and parent-reported parent task involvement scores while CPCC and PPCC are child- and parent-reported Parent–Child conflict scores (CPCC and PPCC are on log scale)

**Table 3:** Posterior means and 95% credible intervals of initial and transition probabilities from the 3-state pHMM applied to the FMOD trial data

	Initial probability	Transition probability					
		Usual-care			Intervention		
		Discordant	Harmonious	Indifferent	Discordant	Harmonious	Indifferent
Discordant	0.50 (0.27, 0.74)	0.58 (0.41, 0.75)	0.37 (0.18, 0.53)	0.05 (0.00, 0.17)	0.48 (0.29, 0.66)	0.47 (0.28, 0.67)	0.05 (0.00, 0.15)
Harmonious	0.44 (0.19, 0.67)	0.03 (0.00, 0.09)	0.47 (0.30, 0.61)	0.50 (0.37, 0.66)	0.03 (0.00, 0.09)	0.65 (0.51, 0.75)	0.32 (0.23, 0.44)
Indifferent	0.07 (0.01, 0.13)	0.01 (0.00, 0.03)	0.01 (0.00, 0.05)	0.98 (0.93, 1.00)	0.02 (0.00, 0.07)	0.02 (0.00, 0.08)	0.96 (0.89, 0.99)

**Table 4:** Posterior means and 95% credible intervals of perception probabilities from the 3-state pHMM applied to the FMOD trial data

Family	Child			Parent		
	Discordant	Harmonious	Indifferent	Discordant	Harmonious	Indifferent
Discordant	0.52 (0.38, 0.69)	0.44 (0.27, 0.58)	0.04 (0.00, 0.13)	0.75 (0.49, 0.97)	0.23 (0.01, 0.49)	0.02 (0.00, 0.07)
Harmonious	0.06 (0.00, 0.18)	0.75 (0.61, 0.91)	0.19 (0.06, 0.32)	0.02 (0.00, 0.07)	0.94 (0.83, 0.99)	0.04 (0.00, 0.13)
Indifferent	0.02 (0.00, 0.06)	0.26 (0.17, 0.35)	0.72 (0.63, 0.81)	0.01 (0.00, 0.04)	0.02 (0.00, 0.07)	0.96 (0.91, 0.99)

**Table 5:** Summaries of the posterior predictive checks of means and variances of the four outcomes based on the 3-state pHMM against the FMOD trial data

Statistic	Quantile					
	2.5%	25%	50%	75%	97.5%	Observed (percentile)
Mean						
CDFR	32.53	32.90	33.09	33.29	33.66	33.05 (0.44)
CPCC	3.18	3.20	3.21	3.22	3.23	3.21 (0.44)
PDFR	35.00	35.37	35.56	35.75	36.11	35.51 (0.43)
PPCC	3.22	3.23	3.24	3.25	3.27	3.24 (0.48)
Variance						
CDFR	18.93	20.61	21.66	22.80	25.22	21.21 (0.39)
CPCC	0.05	0.06	0.06	0.06	0.07	0.05 (0.08)
PDFR	18.29	20.05	21.07	22.18	24.47	21.19 (0.53)
PPCC	0.04	0.05	0.05	0.05	0.06	0.05 (0.18)

CDFR and PDFR are child- and parent-reported parent task involvement scores while CPCC and PPCC are child- and parent-reported Parent–Child conflict scores (CPCC and PPCC are on log scale)
