# Supplementary material for: A Perception-Augmented Hidden Markov Model for Parent–Child Relations in Families of Youth with Type 1 Diabetes
Source: Stat Biosci. Author manuscript; Available in PMC 2025 Aug 27. (PMC12380385; doi:10.1007/s12561-022-09360-8)
Supplement: Supplementary Material [file NIHMS2105517-supplement-Supplementary_Material.pdf]

SUPPLEMENTARY MATERIAL

A An initial look at FMOD trial data

Table A.1: Posterior means (95% credible intervals) of fixed effects of hidden states on means of conditional distributions from the multivariate hidden Markov model with random effects (MHMM). CDFR and PDFR are child- and parent-reported parent task involvement scores while CPCC and PPCC are child- and parent-reported parent-child conflict scores. Family model used pooled data from parents and children while Parent and Child models only used the relevant subset of the data. (Natural logarithm was taken on CPCC and PPCC.)

Outcome	Parent-child relationship		
	Discordant	Harmonious	Indifferent
**Family model**			
CDFR	33.48 (32.30, 34.60)	33.59 (32.58, 34.50)	30.40 (29.44, 31.27)
CPCC	3.30 (3.23, 3.36)	3.14 (3.09, 3.19)	3.11 (3.06, 3.16)
PDFR	38.09 (37.14, 39.06)	36.82 (35.99, 37.73)	33.65 (32.76, 34.61)
PPCC	3.29 (3.24, 3.35)	3.23 (3.19, 3.28)	3.23 (3.19, 3.28)
**Child model**			
CDFR	33.65 (32.35, 34.79)	34.48 (33.42, 35.40)	30.56 (29.51, 31.48)
CPCC	3.31 (3.24, 3.39)	3.16 (3.11, 3.22)	3.06 (3.01, 3.11)
**Parent model**			
PDFR	39.29 (38.00, 40.45)	37.15 (36.29, 38.01)	33.75 (32.89, 34.63)
PPCC	3.47 (3.37, 3.57)	3.21 (3.17, 3.25)	3.22 (3.18, 3.25)

Table A.2: Posterior means (95% credible intervals) of initial and transition probabilities from the multivariate hidden Markov model with random effects (MHMM). CDFR and PDFR are child- and parent-reported parent task involvement scores while CPCC and PPCC are child- and parent-reported parent-child conflict scores. Family model used pooled data from parents and children while Parent and Child models only used the relevant subset of the data.

	Initial probability	Transition probability					
		Usual-care			Intervention		
		Discordant	Harmonious	Indifferent	Discordant	Harmonious	Indifferent
**Family model**							
Discordant	0.43 (0.34, 0.53)	0.50 (0.35, 0.64)	0.45 (0.32, 0.60)	0.05 (0.00, 0.14)	0.37 (0.24,0.52)	0.58 (0.42,0.73)	0.05 (0.00,0.14)
Harmonious	0.49 (0.38, 0.61)	0.07 (0.02, 0.13)	0.60 (0.49, 0.70)	0.33 (0.24, 0.43)	0.08 (0.03,0.13)	0.66 (0.57,0.74)	0.26 (0.19,0.33)
Indifferent	0.08 (0.01, 0.14)	0.01 (0.00, 0.04)	0.01 (0.00, 0.04)	0.98 (0.94, 1.00)	0.03 0.03 (0.00,0.08)	0.02 (0.00,0.06)	0.95 (0.89,0.99)
**Child model**							
Discordant	0.35 (0.27,0.45)	0.35 (0.13, 0.55)	0.45 (0.24, 0.65)	0.20 (0.05, 0.42)	0.23 (0.08,0.41)	0.67 (0.48,0.84)	0.10 (0.01,0.24)
Harmonious	0.44 (0.32,0.55)	0.05 (0.01, 0.10)	0.66 (0.55, 0.75)	0.29 (0.20, 0.40)	0.07 (0.02,0.14)	0.62 (0.5,0.73)	0.31 (0.21,0.42)
Indifferent	0.21 (0.12,0.30)	0.02 (0.00, 0.06)	0.17 (0.09, 0.27)	0.81 (0.72, 0.89)	0.05 (0.01,0.12)	0.16 (0.05,0.29)	0.79 (0.65,0.89)
**Parent model**							
Discordant	0.18 (0.1,0.26)	0.77 (0.63, 0.88)	0.15 (0.03, 0.31)	0.07 (0.00, 0.20)	0.71 (0.54,0.86)	0.23 (0.05,0.41)	0.06 (0.00,0.20)
Harmonious	0.76 (0.65,0.85)	0.02 (0.00, 0.05)	0.49 (0.36, 0.61)	0.49 (0.37, 0.62)	0.02 (0.00, 0.04)	0.66 (0.56,0.75)	0.32 (0.24,0.43)
Indifferent	0.06 (0.01,0.16)	0.01 (0.00, 0.03)	0.02 (0.00, 0.06)	0.97 (0.93, 1.00)	0.01 (0.00,0.03)	0.02 (0.00,0.07)	0.97 (0.92,1.00)

Table A.3: Cross-tabulation frequency (proportion) of estimated latent states obtained by applying MHMM to the parents' and children's subsets of FMOD trial data respectively.

		Child model		
		Discordant	Harmonious	Indifferent
Parent model	Discordant	60 (0.24)	133 (0.52)	61 (0.24)
	Harmonious	141 (0.16)	459 (0.53)	262 (0.30)
	Indifferent	64 (0.08)	337 (0.40)	433 (0.52)

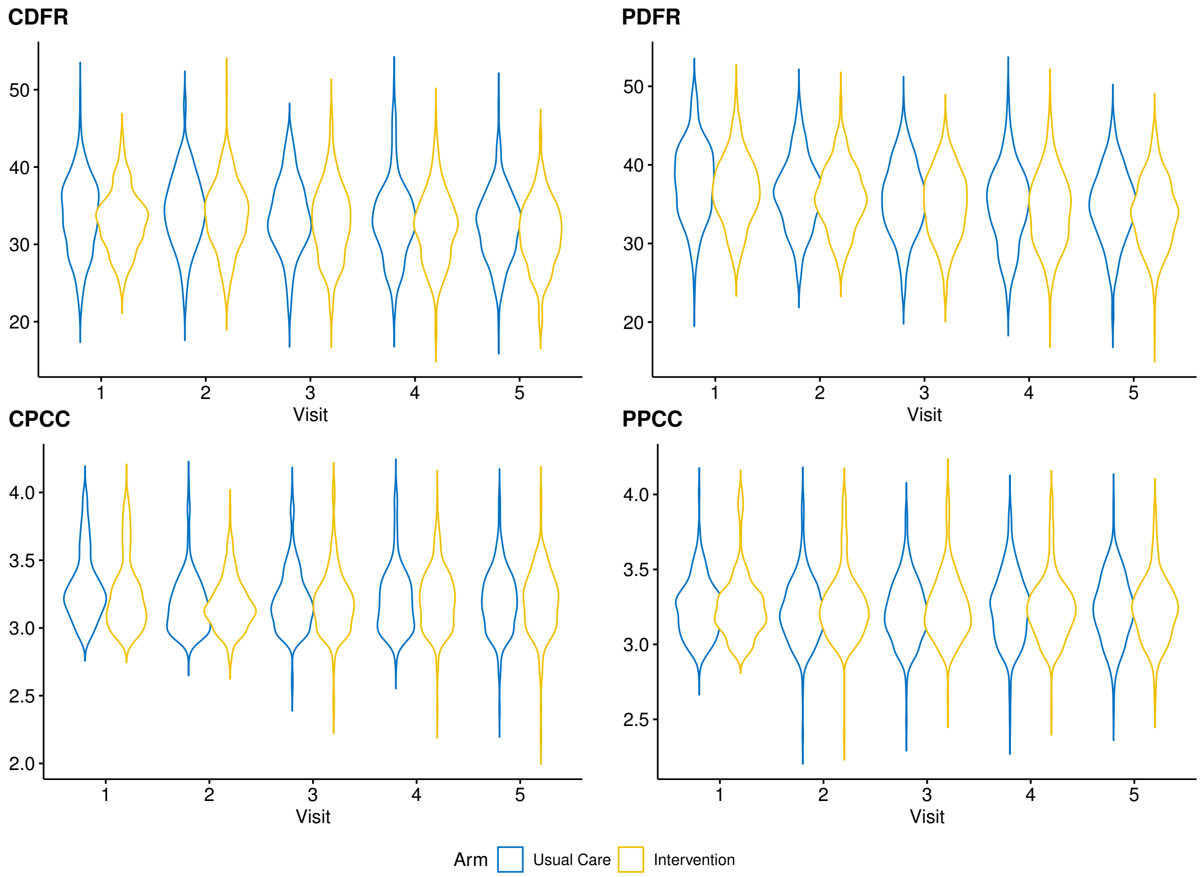

Figure A.1: Violin plots of the four manifesto variables from the FMOD trial data. CDFR and PDFR are child- and parent-reported parent task involvement scores while CPCC and PPCC are child- and parent-reported parent-child conflict scores. Natural logarithm transformation was taken on CPCC and PPCC. CDFR and PDFR are on the original scale.

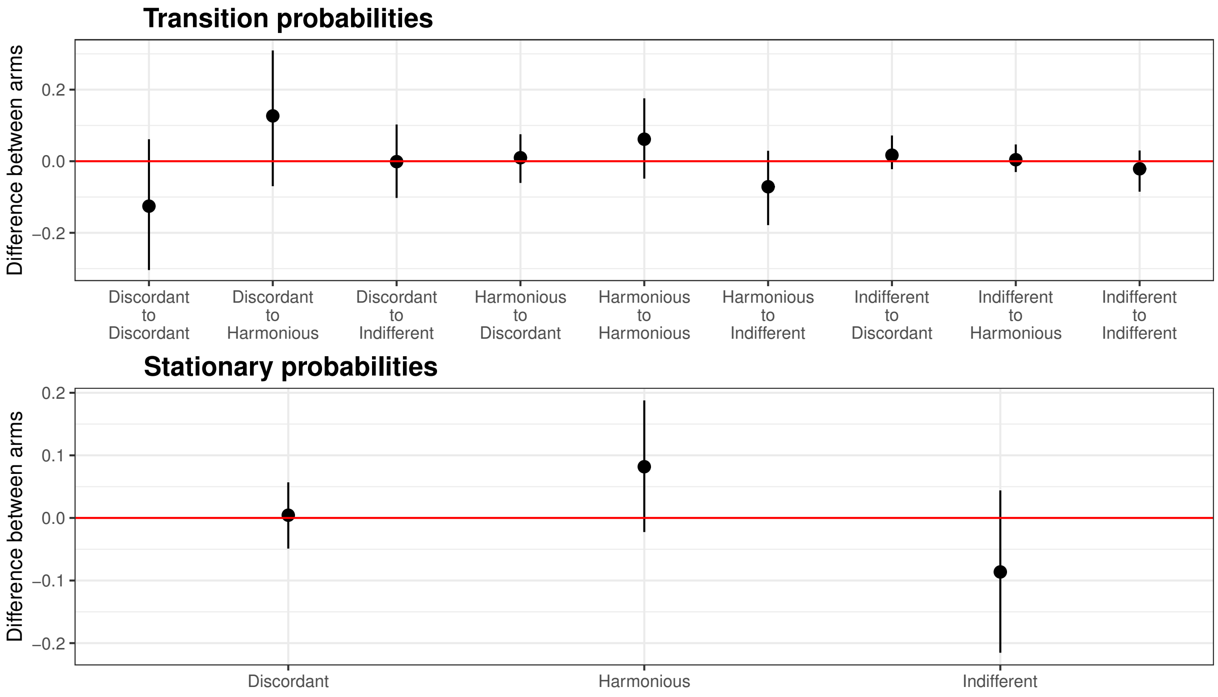

Figure A.2: 95% posterior credible intervals of the average transition probability differences and average stationary probability differences between intervention and usual-care arms from MHMM using pooled data from both parents and children (i.e., Family model).

B Revisit of the FMOD trial data

Table B.1: WAIC of pHMM applied to FMOD trail data with different numbers of latent states for family, parent and child.

Family		2			3			4		
Parent		2	3	4	2	3	4	2	3	4
	2	14963.8	14011.3	13626.3	14924.4	13959.9	13478.6	14931.2	13950.3	13421.9
Child	3	14703.8	13683.6	13251.5	14739.4	13577.2	13129.1	14583.2	13572.7	13151.4
	4	14491.6	13458.4	12947.3	14438.4	13403.5	12934.8	14381.9	13385.4	12926.8

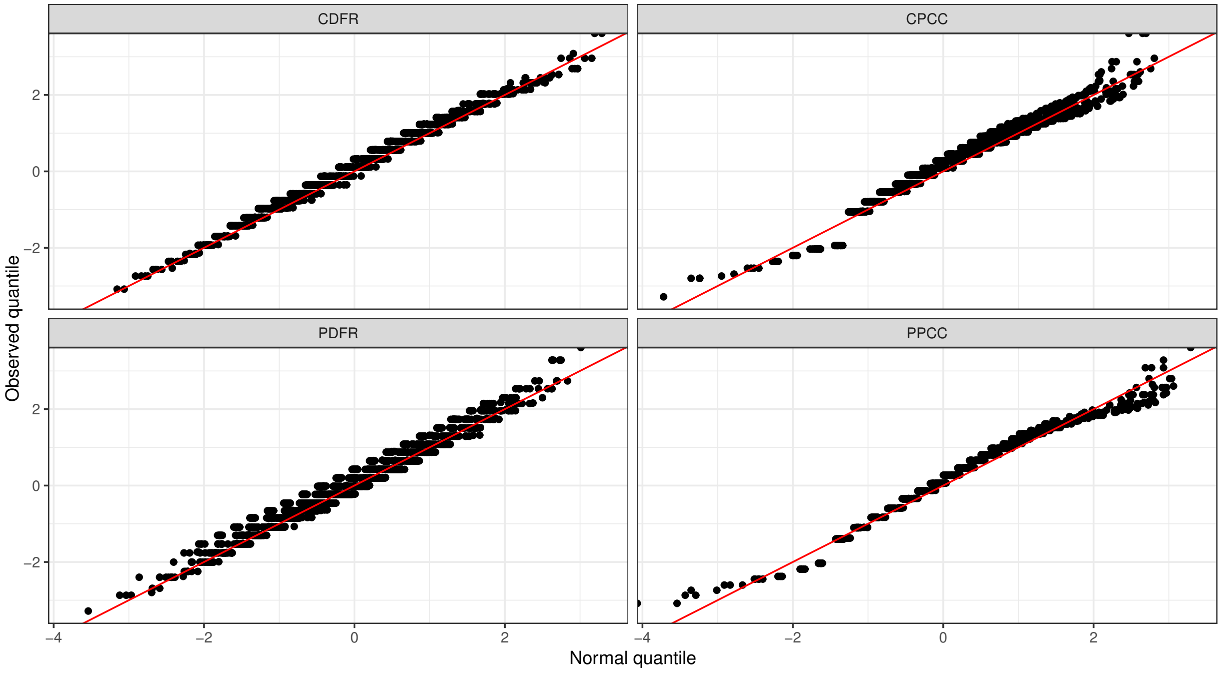

Figure B.1: Normal Q-Q plots of ordinary pseudo-residuals for all four outcomes under the 3-state pHMM applied to the FMOD trial data. CDFR and PDFR are child- and parent-reported parent task involvement scores while CPCC and PPCC are child- and parent-reported parent-child conflict scores. Natural logarithm transformation was taken on CPCC and PPCC.

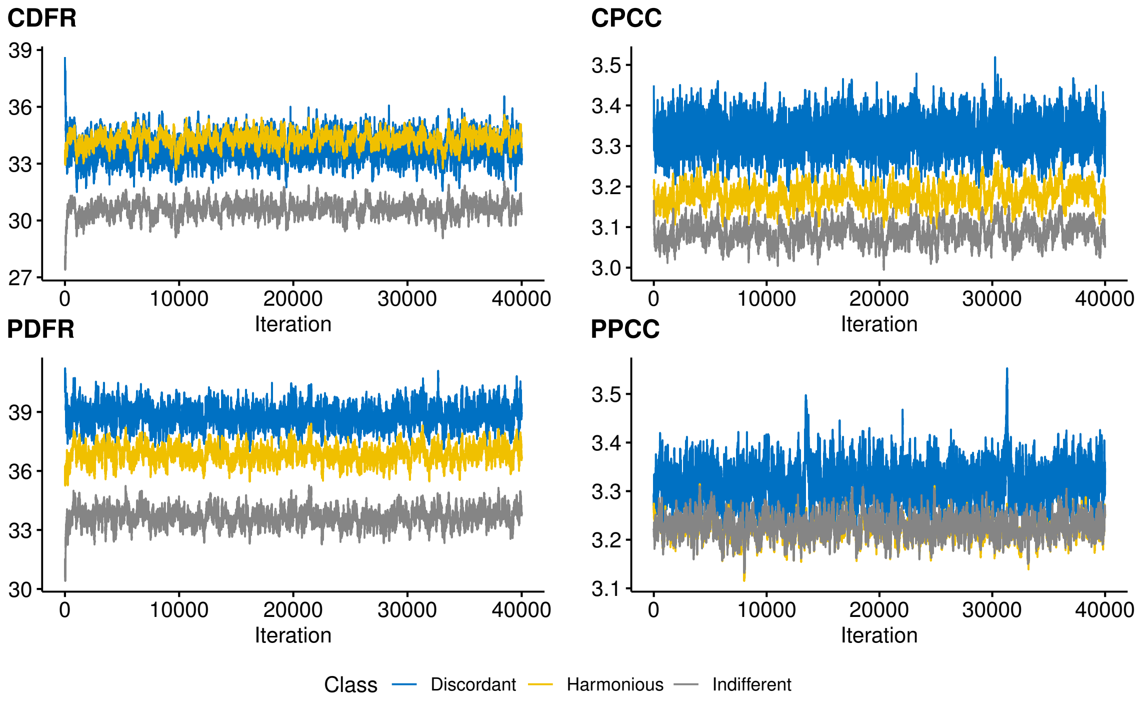

Figure B.2: Trace plots of the fixed effect parameters in the emission model when applying the 3-state pHMM to the FMOD trial data. Different colors stand for different latent states. Natural logarithm transformation was taken on CPCC and PPCC.

C Simulation Studies

Table C.1: WAIC of pHMM with different numbers of hidden states. Data was generated with a 3-state pHMM. The table shows the average value of WAIC over 1000 simulation runs with the standard deviation in the parentheses.

	Number of latent states			
	2	3	4	5
WAIC	15020.10 (187.40)	13437.10 (227.99)	12889.08 (369.81)	12470.30 (355.40)

Table C.2: Simulation results of initial and transition probabilities when MHMM generated the data. Reported are the average values over 1000 simulation runs. "Mean", "SD" and "Rate" correspond to posterior mean, posterior standard deviation, and the proportion of 95% credible interval covering the true value, respectively. $P1$ and $P2$ are transition matrices for usual-care and intervention treatment arms, respectively. The subscripts of P1 and P2 indicate states transiting from and to. π_1 and π_2 are initial probabilities of the first two hidden states.

		MHMM			pHMM			pHMMe		
Parameter	True	Mean	SD	Rate	Mean	SD	Rate	Mean	SD	Rate
π_1	0.441	0.442	0.037	0.943	0.460	0.043	0.936	0.469	0.045	0.917
π_2	0.485	0.473	0.045	0.931	0.459	0.050	0.933	0.450	0.051	0.919
**Usual-care**										
$P1_{12}$	0.456	0.432	0.066	0.942	0.416	0.070	0.923	0.411	0.075	0.932
$P1_{13}$	0.079	0.104	0.046	0.941	0.104	0.049	0.961	0.105	0.052	0.957
$P1_{21}$	0.071	0.076	0.028	0.958	0.061	0.029	0.948	0.055	0.030	0.940
$P1_{23}$	0.327	0.347	0.049	0.940	0.366	0.054	0.896	0.380	0.058	0.862
$P1_{31}$	0.013	0.023	0.015	0.965	0.022	0.015	0.976	0.022	0.015	0.984
$P1_{32}$	0.011	0.032	0.022	0.953	0.030	0.023	0.971	0.030	0.023	0.977
**Intervention**										
$P2_{12}$	0.583	0.554	0.067	0.934	0.541	0.074	0.934	0.530	0.079	0.916
$P2_{13}$	0.045	0.081	0.044	0.928	0.082	0.047	0.947	0.088	0.051	0.938
$P2_{21}$	0.081	0.085	0.026	0.956	0.072	0.028	0.940	0.068	0.030	0.932
$P2_{23}$	0.256	0.277	0.041	0.912	0.291	0.045	0.885	0.305	0.049	0.839
$P2_{31}$	0.007	0.021	0.015	0.957	0.021	0.016	0.968	0.021	0.016	0.970
$P2_{32}$	0.012	0.035	0.025	0.964	0.034	0.026	0.982	0.034	0.026	0.986

Table C.3: Simulation results of perception matrices when MHMM generated the data. Reported are the average value of 1000 simulation runs. "Mean" and "SD" are corresponding to posterior mean and posterior standard deviation, respectively. "SE" in the parentheses gives the standard deviation of posterior means over the simulation runs. P_m denotes perception matrix. Its superscripts stand for the member and the subscripts indicate the hidden states. Note that MHMM does not estimate P_m directly. The results are estimated by cross-tabulating the estimated latent classes with separate models. Constrained framework has the emission model of parent and child fixed as estimation results from Family model. Unconstrained framework has separate models for family, parent and child.

		MHMM		pHMM		pHMMe			
		Constrained	Unconstrained			Usual-care		Intervention	
Parameter	True	Mean (SE)	Mean (SE)	Mean	SD	Mean	SD	Mean	SD
**Child**									
$Pm_{11}^{(1)}$	1	0.798 (0.03)	0.792 (0.07)	0.893	0.054	0.858	0.068	0.847	0.073
$Pm_{21}^{(1)}$	0	0.101 (0.03)	0.109 (0.06)	0.022	0.017	0.035	0.028	0.030	0.024
$Pm_{31}^{(1)}$	0	0.088 (0.02)	0.089 (0.03)	0.009	0.008	0.014	0.012	0.015	0.013
$Pm_{12}^{(1)}$	0	0.146 (0.03)	0.146 (0.05)	0.066	0.047	0.086	0.060	0.091	0.063
$Pm_{22}^{(1)}$	1	0.750 (0.04)	0.720 (0.08)	0.935	0.036	0.900	0.054	0.910	0.049
$Pm_{32}^{(1)}$	0	0.213 (0.04)	0.193 (0.06)	0.040	0.029	0.052	0.037	0.058	0.041
**Parent**									
$Pm_{11}^{(2)}$	1	0.630 (0.05)	0.607 (0.01)	0.888	0.059	0.850	0.075	0.848	0.077
$Pm_{21}^{(2)}$	0	0.148 (0.03)	0.228 (0.17)	0.029	0.023	0.047	0.036	0.041	0.032
$Pm_{31}^{(2)}$	0	0.094 (0.03)	0.110 (0.05)	0.012	0.010	0.017	0.015	0.020	0.017
$Pm_{12}^{(2)}$	0	0.291 (0.04)	0.308 (0.13)	0.076	0.054	0.099	0.068	0.099	0.070
$Pm_{22}^{(2)}$	1	0.710 (0.04)	0.612 (0.17)	0.931	0.038	0.892	0.055	0.903	0.050
$Pm_{32}^{(2)}$	0	0.208 (0.03)	0.181 (0.06)	0.045	0.032	0.059	0.041	0.066	0.045

Table C.4: Simulation results of initial and transition probabilities when pHMM generated the data. Reported are the average values over 1000 simulation runs. “Mean”, “SD” and “Rate” correspond to posterior mean, posterior standard deviation, and the proportion of 95% credible interval covering the true value, respectively. $P1$ and $P2$ are transition matrices for usual-care and intervention treatment arms, respectively. The subscripts of P1 and P2 indicate states transiting from and to. π_1 and π_2 are initial probabilities of the first two hidden states.

		pHMM			MHMM			pHMMe		
Parameter	True	Mean	SD	Rate	Mean	SD	Rate	Mean	SD	Rate
π_1	0.760	0.778	0.083	0.970	0.127	0.018	0.001	0.758	0.087	0.970
π_2	0.180	0.158	0.083	0.964	0.781	0.044	0.001	0.176	0.088	0.961
**Usual-care**										
$P1_{12}$	0.702	0.699	0.088	0.982	0.672	0.120	0.944	0.701	0.093	0.975
$P1_{13}$	0.073	0.098	0.050	0.966	0.249	0.115	0.690	0.104	0.055	0.962
$P1_{21}$	0.024	0.046	0.035	0.987	0.024	0.009	0.968	0.047	0.036	0.991
$P1_{23}$	0.240	0.268	0.048	0.930	0.268	0.042	0.880	0.277	0.054	0.920
$P1_{31}$	0.014	0.030	0.024	0.993	0.010	0.007	0.974	0.027	0.023	0.996
$P1_{32}$	0.020	0.052	0.039	0.986	0.037	0.024	0.973	0.050	0.039	0.994
**Intervention**										
$P2_{12}$	0.764	0.736	0.087	0.972	0.694	0.117	0.918	0.752	0.090	0.990
$P2_{13}$	0.049	0.080	0.046	0.968	0.231	0.112	0.641	0.080	0.049	0.974
$P2_{21}$	0.049	0.061	0.042	0.994	0.025	0.009	0.339	0.055	0.040	0.993
$P2_{23}$	0.369	0.368	0.059	0.955	0.320	0.043	0.755	0.373	0.062	0.960
$P2_{31}$	0.013	0.027	0.021	0.992	0.010	0.007	0.976	0.025	0.020	0.993
$P2_{32}$	0.021	0.046	0.034	0.992	0.032	0.021	0.966	0.044	0.034	0.994

Table C.5: Simulation results of perception matrices when pHMM generated the data. Reported are the average value of 1000 simulation runs. "Mean" and "SD" are corresponding to posterior mean and posterior standard deviation, respectively. "SE" in the parentheses gives the standard deviation of posterior means over the simulation runs. "Rate" corresponds the proportion of 95% credible interval covering the true value. P_m denotes perception matrix. Its superscripts stand for the member and the subscripts indicate the hidden states. Note that MHMM does not estimate P_m directly. The results are estimated by cross-tabulating the estimated latent states with separate models. Constrained framework has the emission model of parent and child fixed as estimation results from Family model. Unconstrained framework has separate models for family, parent and child.

		pHMM			MHMM		pHMMe					
					Unconstrained	Constrained	Usual-care			Intervention		
Parameter	True	Mean	SD	Rate	Mean (SE)	Mean(SE)	Mean	SD	Rate	Mean	SD	Rate
**Child**												
$Pm_{11}^{(1)}$	0.531	0.521	0.071	0.957	0.589 (0.06)	0.632 (0.05)	0.516	0.089	0.943	0.517	0.094	0.950
$Pm_{21}^{(1)}$	0.051	0.056	0.031	0.987	0.046 (0.05)	0.056 (0.02)	0.086	0.050	0.967	0.074	0.042	0.971
$Pm_{31}^{(1)}$	0.025	0.028	0.013	0.968	0.017 (0.01)	0.026 (0.02)	0.031	0.017	0.973	0.034	0.020	0.976
$Pm_{12}^{(1)}$	0.433	0.416	0.071	0.954	0.276 (0.06)	0.264 (0.05)	0.401	0.093	0.927	0.393	0.098	0.944
$Pm_{22}^{(1)}$	0.744	0.748	0.058	0.954	0.752 (0.08)	0.757 (0.04)	0.733	0.082	0.945	0.737	0.073	0.951
$Pm_{32}^{(1)}$	0.253	0.251	0.044	0.948	0.257 (0.07)	0.225 (0.06)	0.247	0.051	0.952	0.254	0.060	0.943
**Parent**												
$Pm_{11}^{(2)}$	0.740	0.721	0.089	0.959	0.590 (0.07)	0.585 (0.05)	0.704	0.102	0.948	0.696	0.106	0.957
$Pm_{21}^{(2)}$	0.021	0.050	0.036	0.983	0.157 (0.08)	0.133 (0.04)	0.100	0.063	0.929	0.078	0.050	0.937
$Pm_{31}^{(2)}$	0.015	0.016	0.011	0.992	0.053 (0.03)	0.076 (0.03)	0.021	0.014	0.994	0.022	0.016	0.995
$Pm_{12}^{(2)}$	0.238	0.234	0.091	0.962	0.297 (0.07)	0.310 (0.04)	0.237	0.102	0.955	0.239	0.107	0.963
$Pm_{22}^{(2)}$	0.942	0.896	0.052	0.978	0.693 (0.09)	0.716 (0.05)	0.820	0.081	0.851	0.854	0.068	0.890
$Pm_{32}^{(2)}$	0.023	0.048	0.032	0.979	0.213 (0.06)	0.205 (0.05)	0.059	0.038	0.967	0.073	0.048	0.954

Table C.6: Simulation results of initial and transition probabilities when pHMMe generated the data. Reported are the average values over 1000 simulation runs. "Mean", "SD" and "Rate" correspond to posterior mean, posterior standard deviation, and the proportion of 95% credible interval covering the true value, respectively. $P1$ and $P2$ are transition matrices for usual-care and intervention treatment arms, respectively. The subscripts of P1 and P2 indicate states transiting from and to. π_1 and π_2 are initial probabilities of the first two hidden states.

		pHMMe			MHMM			pHMM		
Parameter	True	Mean	SD	Rate	Mean	SD	Rate	Mean	SD	Rate
π_1	0.495	0.521	0.057	0.940	0.443	0.040	0.731	0.504	0.061	0.970
π_2	0.437	0.398	0.061	0.923	0.474	0.047	0.821	0.415	0.065	0.957
**Usual-care**										
$P1_{12}$	0.379	0.404	0.095	0.959	0.525	0.075	0.507	0.477	0.087	0.824
$P1_{13}$	0.047	0.093	0.056	0.971	0.101	0.050	0.868	0.068	0.046	0.991
$P1_{21}$	0.029	0.073	0.052	0.975	0.114	0.035	0.224	0.065	0.040	0.939
$P1_{23}$	0.506	0.552	0.092	0.943	0.397	0.054	0.503	0.466	0.072	0.894
$P1_{31}$	0.010	0.018	0.014	0.997	0.031	0.018	0.842	0.020	0.015	0.991
$P1_{32}$	0.014	0.036	0.026	0.988	0.101	0.043	0.469	0.050	0.033	0.922
**Intervention**										
$P2_{12}$	0.465	0.428	0.078	0.950	0.408	0.069	0.857	0.368	0.081	0.801
$P2_{13}$	0.048	0.097	0.050	0.911	0.153	0.052	0.437	0.124	0.057	0.788
$P2_{21}$	0.026	0.043	0.030	0.988	0.107	0.036	0.280	0.050	0.036	0.985
$P2_{23}$	0.323	0.385	0.063	0.839	0.362	0.056	0.894	0.415	0.072	0.742
$P2_{31}$	0.020	0.023	0.015	0.993	0.030	0.017	0.962	0.023	0.015	0.996
$P2_{32}$	0.022	0.041	0.028	0.984	0.050	0.029	0.929	0.033	0.025	0.996

Table C.7: Simulation results of perception matrices when pHMMe generated the data. Reported are the average value of 1000 simulation runs. "Mean" and "SD" are corresponding to posterior mean and posterior standard deviation, respectively. "SE" in the parentheses gives the standard deviation of posterior means over the simulation runs. "Rate" corresponds the proportion of 95% credible interval covering the true value. P_m denotes perception matrix. Its superscripts stand for the member and the subscripts indicate the hidden states. Note that MHMM does not estimate P_m directly. The results are estimated by cross-tabulating the estimated latent states with separate models. Constrained framework has the emission model of parent and child fixed as estimation results from Family model. Unconstrained framework has separate models for family, parent and child.

Parameter	pHMMe								MHMM		pHMM	
	Usual-care				Intervention				Unconstrained	Constrained		
	True	Mean	SD	Rate	True	Mean	SD	Rate	Mean (SE)	Mean (SE)	Mean	SD
**Child**												
$Pm_{11}^{(1)}$	0.531	0.504	0.082	0.937	0.827	0.784	0.071	0.959	0.682 (0.05)	0.697 (0.04)	0.681	0.071
$Pm_{32}^{(1)}$	0.051	0.077	0.048	0.978	0.133	0.124	0.050	0.954	0.067 (0.02)	0.070 (0.02)	0.087	0.039
$Pm_{31}^{(1)}$	0.025	0.031	0.017	0.974	0	0.018	0.014	-	0.012 (0.01)	0.014 (0.01)	0.021	0.012
$Pm_{12}^{(1)}$	0.433	0.418	0.086	0.950	0.039	0.085	0.054	0.972	0.210 (0.04)	0.229 (0.04)	0.217	0.069
$Pm_{22}^{(1)}$	0.744	0.747	0.081	0.949	0.487	0.507	0.074	0.934	0.631 (0.08)	0.697 (0.05)	0.643	0.068
$Pm_{32}^{(1)}$	0.253	0.259	0.051	0.951	0.009	0.040	0.025	0.933	0.152 (0.07)	0.157 (0.05)	0.131	0.039
**Parent**												
$Pm_{11}^{(2)}$	0.740	0.698	0.098	0.945	0.887	0.791	0.079	0.850	0.605 (0.07)	0.619 (0.04)	0.776	0.079
$Pm_{21}^{(2)}$	0.021	0.087	0.057	0.955	0.001	0.039	0.032	0.445	0.154 (0.06)	0.156 (0.04)	0.045	0.034
$Pm_{31}^{(2)}$	0.015	0.021	0.015	0.994	0	0.013	0.011	-	0.066 (0.03)	0.091 (0.03)	0.011	0.008
$Pm_{12}^{(2)}$	0.238	0.246	0.098	0.961	0.028	0.113	0.073	0.934	0.280 (0.06)	0.283 (0.04)	0.157	0.078
$Pm_{22}^{(2)}$	0.942	0.831	0.078	0.878	0.970	0.860	0.062	0.601	0.664 (0.08)	0.684 (0.05)	0.880	0.056
$Pm_{32}^{(2)}$	0.023	0.060	0.039	0.971	0.031	0.077	0.046	0.947	0.215 (0.06)	0.226 (0.05)	0.052	0.033

Table C.8: Simulation results of estimating hidden states. Precision, recall, F-score, accuracy and error rate are class-wise average of the corresponding criteria. Precision$_m$ stands for the micro average version of precision. The average values of criteria over 1000 simulation runs are reported with the standard deviations in the parentheses.

Metric	MHMM	pHMM			pHMMe		
	Family	Family	Child	Parent	Family	Child	Parent
**Scenario 1**							
Precision	0.767 (0.037)	0.740 (0.065)	-	-	0.727 (0.065)	-	-
Recall	0.760 (0.036)	0.748 (0.065)	-	-	0.741 (0.065)	-	-
F-score	0.764 (0.036)	0.744 (0.065)	-	-	0.734 (0.065)	-	-
Accuracy	0.842 (0.027)	0.828 (0.046)	-	-	0.822 (0.045)	-	-
Err. rate	0.158 (0.027)	0.172 (0.046)	-	-	0.178 (0.045)	-	-
Precision$_m$	0.762 (0.041)	0.743 (0.069)	-	-	0.733 (0.067)	-	-
**Scenario 2**							
Precision	0.683 (0.034)	0.684 (0.020)	0.807 (0.017)	0.732 (0.020)	0.670 (0.040)	0.803 (0.018)	0.728 (0.020)
Recall	0.651 (0.033)	0.693 (0.019)	0.745 (0.018)	0.711 (0.018)	0.680 (0.039)	0.745 (0.017)	0.709 (0.019)
F-score	0.667 (0.033)	0.688 (0.019)	0.774 (0.012)	0.721 (0.014)	0.675 (0.039)	0.773 (0.012)	0.718 (0.015)
Accuracy	0.781 (0.023)	0.794 (0.014)	0.860 (0.009)	0.821 (0.010)	0.786 (0.025)	0.859 (0.009)	0.820 (0.010)
Err. rate	0.219 (0.023)	0.206 (0.014)	0.140 (0.009)	0.179 (0.010)	0.214 (0.025)	0.141 (0.009)	0.180 (0.010)
Precision$_m$	0.672 (0.034)	0.691 (0.021)	0.790 (0.013)	0.732 (0.015)	0.680 (0.037)	0.789 (0.013)	0.730 (0.015)
**Scenario 3**							
Precision	0.705 (0.031)	0.695 (0.023)	0.771 (0.015)	0.735 (0.017)	0.698 (0.032)	0.799 (0.013)	0.743 (0.021)
Recall	0.689 (0.032)	0.706 (0.021)	0.752 (0.016)	0.726 (0.018)	0.712 (0.029)	0.776 (0.014)	0.734 (0.020)
F-score	0.697 (0.031)	0.700 (0.022)	0.762 (0.013)	0.731 (0.015)	0.705 (0.031)	0.788 (0.012)	0.739 (0.018)
Accuracy	0.800 (0.024)	0.801 (0.016)	0.852 (0.009)	0.826 (0.010)	0.805 (0.021)	0.868 (0.008)	0.832 (0.012)
Err. Rate	0.200 (0.024)	0.199 (0.016)	0.148 (0.009)	0.174 (0.010)	0.195 (0.021)	0.132 (0.008)	0.168 (0.012)
Precision$_m$	0.699 (0.036)	0.702 (0.024)	0.777 (0.014)	0.739 (0.015)	0.707 (0.032)	0.802 (0.012)	0.748 (0.018)
